# The dangerous link between coal dust exposure and DNA damage: unraveling the role of some of the chemical agents and oxidative stress

**DOI:** 10.1007/s10653-023-01697-3

**Published:** 2023-08-04

**Authors:** Alvaro Miranda-Guevara, Amner Muñoz-Acevedo, Ornella Fiorillo-Moreno, Antonio Acosta-Hoyos, Leonardo Pacheco-Londoño, Milton Quintana-Sosa, Yurina De Moya, Johnny Dias, Guilherme Soares de Souza, Wilner Martinez-Lopez, Ana Letícia Hilário Garcia, Juliana da Silva, Malu Siqueira Borges, João Antonio Pêgas Henriques, Grethel León-Mejía

**Affiliations:** 1 Centro de Investigaciones en Ciencias de la Vida (CICV), Universidad Simón Bolívar, Cra 53 Calle 64-51, Barranquilla, 080002 Colombia; 2 Grupo de Investigación en Química y Biología, Universidad del Norte, Barranquilla, Colombia; 3 Clínica Iberoamerica, Barranquilla, Colombia; 4 Clinica el Carmen, Barranquilla, Colombia; 5 Laboratório de Implantação Iônica, Instituto de Física, Universidade Federal do Rio Grande do Sul (UFRGS), Porto Alegre, RS Brazil; 6 Ministry of Education and Culture, Instituto de Investigaciones Biológicas Clemente Estable, Montevideo, Uruguay; 7 Laboratory of Genetic Toxicology, La Salle University (UniLaSalle), Canoas, RS Brazil; 8 Laboratory of Genetic Toxicology. PPGBioSaúde (Postgraduate Program in Cellular and Molecular Biology Applied to Health), Lutheran University of Brazil (ULBRA), Canoas, RS Brazil; 9 Departamento de Biofísica, Centro de Biotecnologia, Universidade Federal do Rio Grande do Sul (UFRGS), Porto Alegre, RS Brazil; 10 Programa de Pós-Graduação em Biotecnologia e em Ciências Médicas, Universidade do Vale do Taquari - UNIVATES, Lajeado, RS Brazil

**Keywords:** DNA oxidative damage, Telomere length, FPG-modified comet assay, Coal mining

## Abstract

Exposure to coal mining dust poses a substantial health hazard to individuals due to the complex mixture of components released during the extraction process. This study aimed to assess the oxidative potential of residual coal mining dust on human lymphocyte DNA and telomeres and to perform a chemical characterization of coal dust and urine samples. The study included 150 individuals exposed to coal dust for over ten years, along with 120 control individuals. The results revealed significantly higher levels of DNA damage in the exposed group, as indicated by the standard comet assay, and oxidative damage, as determined by the FPG-modified comet assay. Moreover, the exposed individuals exhibited significantly shorter telomeres compared to the control group, and a significant correlation was found between telomere length and oxidative DNA damage. Using the PIXE method on urine samples, significantly higher concentrations of sodium (Na), phosphorus (P), sulfur (S), chlorine (Cl), potassium (K), iron (Fe), zinc (Zn), and bromine (Br) were observed in the exposed group compared to the control group. Furthermore, men showed shorter telomeres, greater DNA damage, and higher concentrations of nickel (Ni), calcium (Ca), and chromium (Cr) compared to exposed women. Additionally, the study characterized the particles released into the environment through GC–MS analysis, identifying several compounds, including polycyclic aromatic hydrocarbons (PAHs) such as fluoranthene, naphthalene, anthracene, 7H-benzo[c]fluorene, phenanthrene, pyrene, benz[a]anthracene, chrysene, and some alkyl derivatives. These findings underscore the significant health risks associated with exposure to coal mining dust, emphasizing the importance of further research and the implementation of regulatory measures to safeguard the health of individuals in affected populations.

**Graphic abstract:**

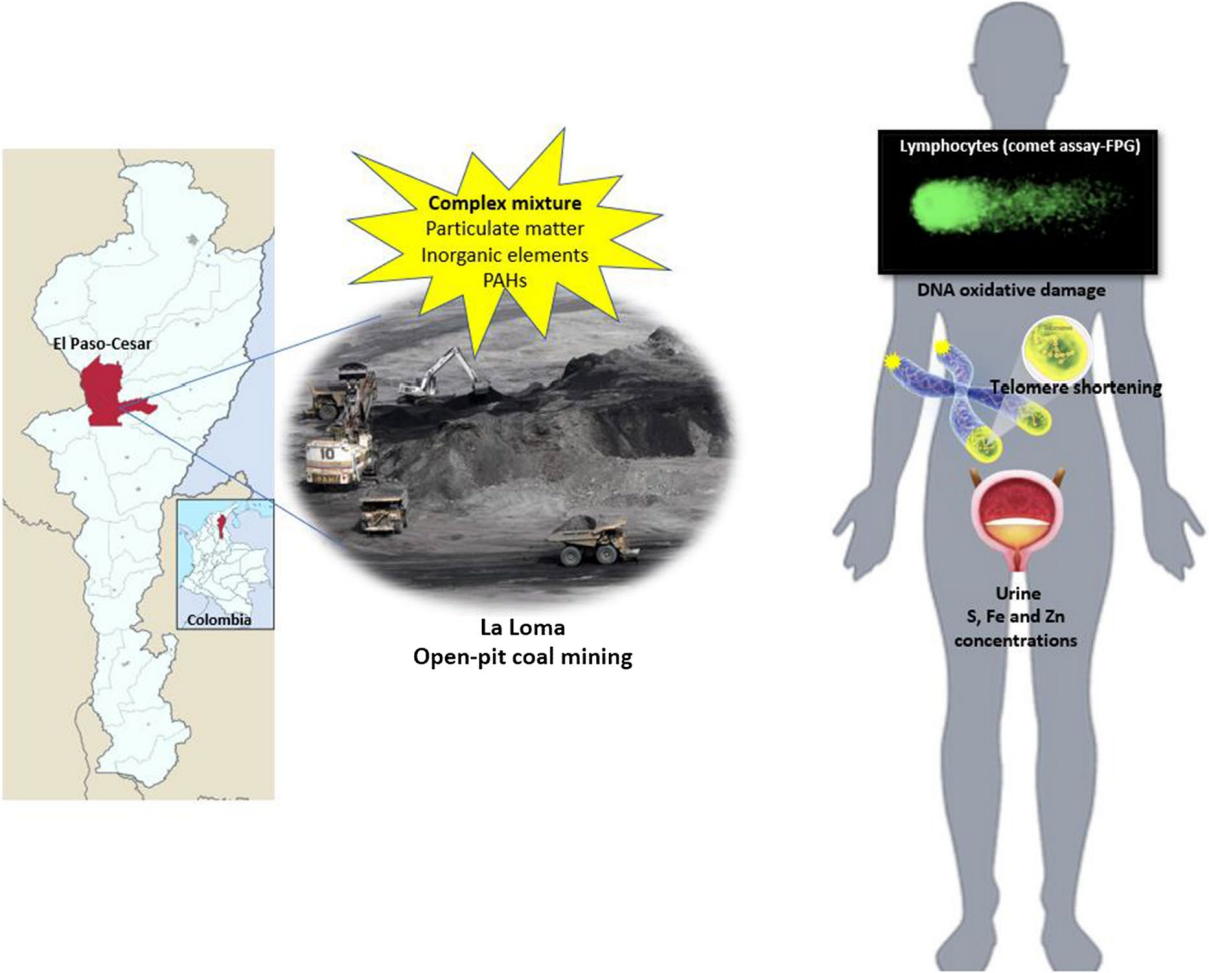

## Introduction

Since the twentieth century, the exploitation and consumption of fossil fuels, particularly coal, have been a key driver of global economic development for industrial and domestic energy generation. However, the combustion of fossil fuels is a significant contributor to climate change due to the release of polluting emissions (Jakob et al., 2020). Coal pollution, including residual dust, is known to contaminate the air, water, and soil (Romana et al., 2022; Hendryx et al., 2020), resulting in an increased risk of heart and respiratory diseases such as asthma, pneumoconiosis and bronchitis, as well as cancer, leading to thousands of premature deaths annually (Song et al., 2022; Kamanzi et al., 2023; Kravchenko & Lyerly, 2018).

In this context, the release of particulate matter into the environment constitutes a highly intricate mixture comprising particles of diverse sizes. Some of these particles, particularly those with a size of < 10 µm known as fine particulate matter (PM), can penetrate the pulmonary alveoli and enter the bloodstream, thereby exerting an impact on extra-pulmonary organs (Hendryx et al., 2020; Song et al., 2022). While numerous studies have examined occupational exposure to coal mining residues (Kvitko et al., 2012; León-Mejía et al., 2011, 2014; Rohr et al., 2013; Sinitsky et al., 2016), only a limited number have focused on populations residing in the surrounding areas who are also exposed. Therefore, biomonitoring studies play a crucial role in establishing the relationship between environmental factors and diseases as they enable the detection of initial alterations in non-malignant phases (Bocato et al., 2019; Lum et al., 2021).

The comet assay is currently one of the most used methodologies for assessing DNA breaks and estimating damage in individual cells (Azqueta et al., 2020). To detect oxidative damage, enzyme treatments have been incorporated, such as the use of formamidopyrimidine DNA glycosylase (FPG) to detect oxidized purine bases, including 8-oxo guanine and 4,6-diamino-5-formamidopyrimidine (Fapy-Ade) lesions (Muruzabal et al., 2021). This approach is suitable for analyzing the extent of DNA damage induced indirectly by reactive oxygen species (ROS) resulting from oxidative stress (Balasubramanyam et al., 2010); Kuchařová et al., 2019).

On the other hand, the measurement of telomeres can serve as a complementary monitoring tool and provide additional insights into the effects of xenobiotics on cells and the generation of oxidative stress in individuals. Critically shortened telomeres can trigger cellular senescence in normal cells or genomic instability in premalignant cells, thereby contributing to the development of chronic diseases, including degenerative diseases and cancer (Barnes et al., 2019; De Rosa et al., 2021). Therefore, telomere measurements hold promise as biomarkers of exposure to agents that can induce DNA damage and can assist in the risk assessment of such agents (Azqueta et al., 2020).

The study aimed to evaluate the effects of coal mining particles, released in the surrounding area of the town of "La Loma" in Colombia, on the oxidative damage and telomere shortening of human lymphocyte DNA, as well as to determine the organo-inorganic chemical composition of this particulate matter and urine samples from the population under study.

## Materials and methods

### Individuals and sampling

This study was conducted in the mining zone of La Loma-El Paso, located in the Department of Cesar, Colombia, where four open-pit coal mines are present. The sample size comprised 150 individuals (89 women, 61 men) from the mining region who were exposed to coal mining particles, and 120 control individuals (70 women, 50 men) from Barranquilla (Colombia). The exposed individuals reside 2 km away from the nearest coal mine, and additional information can be found in León-Mejía et al., (2023a, 2023b).

The exposed group was matched with the control group based on age (± 2 years), and all individuals completed a survey on socio-demographic information. The survey included questions about lifestyle, occupation, alcohol and meat consumption, vitamin and medication use, family history of cancer, recent exposure to X-rays or other carcinogens, and smoking habits, among others. The exposed group was selected based on voluntary acceptance and having lived in the vicinity of the mining area for at least five years. After being selected, all individuals (exposed and control) provided informed consent. This study was approved by the Ethics Committee of Universidad Simón Bolívar (CIE-USB-CE-0233-00). All the information was kept at the “Centro de Investigaciones en Ciencias de la Vida (CICV), Universidad Simón Bolívar”, Barranquilla, Colombia.

### Collection of blood and urine samples

Blood samples were collected from individuals via venipuncture into heparin and EDTA vacutainers, while urine samples were collected in 30 mL polypropylene containers with lids. The blood and urine samples were collected between 8 and 10 am. We ensured that individuals had not consumed alcohol for at least 24 h before blood collection. All samples were labeled with unique codes. The blood and urine samples were transported from the collection site (Loma-Cesar) to the laboratory at 4 °C. The DNA was extracted the following day and stored along with the urine at − 20 °C for two months until analysis.

### Comet assay

The standard alkaline comet assay, as described by Singh et al., (1988) and Tice et al. (2000), with modifications from León-Mejía et al., (2011, 2016), was conducted. Lymphocytes were isolated using ficoll-histopaque® and combined with 300 µL of low melting point agarose (LMA) at 37 °C. The mixture was then placed on a slide coated with 1.5% normal melting point agarose (NMA) at 60 °C. A coverslip was applied, and the slide was immersed in a lysing solution consisting of 2.5 M NaCl, 100 mM EDTA, and 10 mM Tris (pH 10.0–10.5), with freshly added 1% Triton X-100 and 10% DMSO. The samples were stored in the dark at 4 °C. Next, an unwinding process was performed in an alkaline buffer (300 mM NaOH/1 mM EDTA, pH > 13) for 30 min at 4 °C. The gels were then subjected to alkaline electrophoresis at 25 V and 300 mA for 30 min, followed by neutralization (0.4 M Tris, pH 7.5) and washing (three times for 5 min each). Finally, the slides were stained with 30 μL Sybr Safe (2 μL/mL) and examined under a fluorescence microscope (Zeiss AXIO SCOPE A1 503 model) equipped with a green filter of 540 nm at 40X magnification.

*The FPG-modified comet assay* was performed by adding an FPG enzyme to identify oxidative damage to DNA. The methodology for this assay was like the standard comet assay but with a few modifications. After immersion in the lysis solution, the slides were washed with enzyme buffer three times and then incubated at 37 °C with enzyme buffer supplemented with 60 μL of FPG (1 μg/mL solution) for 45 min. The rest of the steps were the same as in the standard comet assay. Six slides were analyzed per individual, three for the standard comet test and three for the FPG-comet slides. The visual score values ranged from 0 (100 cells, class 0) to 400 (100 cells, class 4). The mean values of all comet assay parameters were considered for statistical analysis.

### Quantitative polymerase chain reaction (qPCR) for the measurement of telomere length (TL)

The DNA samples were assessed for concentration using the NanoDrop 1000 spectrophotometer and then diluted to meet the experimental requirement (5 ng/μL). To determine TL, the procedure outlined by O’Callaghan and Fenech 2011 with minor modifications by Kahl et al. (2016) was followed. A standard curve was generated by creating serial dilutions of a known quantity of a pooled DNA sample. The amplification control involved the single copy gene 36B4, responsible for encoding the acidic ribosomal phosphoprotein PO. Each sample was analyzed in triplicate using the Step One Plus™ Real-Time PCR System (Applied Biosystems, Foster City, CA, USA), with negative and positive controls and a standard curve. A master mix was prepared using SYBR Green PCR Master Mix Power UP (Applied Biosystems, Foster City, CA, USA), 20 ng DNA, injection water, and 0.2 μmol of telomere primers (forward: 5′- CGGTTTGTTTGGGTTTGGGTTTGGGTTTGGGTTTGGGTT -3´; reverse: 5′- GGCTTGCCTTACCCTTACCCTTACCCTTACCCTTACCCT -3′), and 0.2 μmol of 36B4 primers (forward: 5′-CAGCAAGTGGGAAGGTGTAATCC-3'; reverse: 5′-CCCATTCTATCATCAACGGGTACAA -3′). The qPCR was performed using the following parameters for both telomere and 36B4 amplicons: initial activation of Taq polymerase at 95 °C for 10 min, followed by 40 cycles of denaturation at 95 °C for 15 s and annealing plus extension at 60 °C for 1 min. The cycle threshold (Ct) value obtained for each sample was utilized to calculate the total telomere length in kilobases (kb) per human diploid genome.

### Analysis by particle-induced X-ray emission (PIXE)

For the determination of inorganic elements in urine, filter paper (0.45 µm- millipore) and a vacuum filtration system with a 500 mL bottle (Corning) were used. Individual samples from each participant included in the study were filtered and dried at 40 °C for 48 h. Then, each sample was placed inside the reaction chamber at reduced pressure (ca. 10^–6^ mbar), where proton beams (2.0 MeV, average current: 1 nA) delivered by the Tandetron accelerator (3 MV) produced the inner shell ionization of the atoms present in the sample (beam spot size: 4 mm^2^). An electron flood gun was used during the experiment to avoid charge effects on the sample (Shubeita et al., 2005).

The GUPIXWIN® software, developed at the University of Guelph (Canada) by Campbell et al. (2000), was employed to analyze each X-ray spectrum obtained. This software converts the measured peak areas into elemental concentrations. In the urine samples, the elements detected included sodium (Na), phosphorus (P), sulfur (S), chlorine (Cl), potassium (K), calcium (Ca), chromium (Cr), iron (Fe), nickel (Ni), zinc (Zn), and bromine (Br). The Limit of Detection (LOD) for elemental analysis using PIXE varies depending on the specific element and the matrix being analyzed. Elements with lower atomic numbers (*Z*) tend to have relatively higher LODs compared to those with higher atomic numbers (*Z* > 20). The PIXE setup at the Ion Implantation Laboratory is standardized with NIST (National Institute of Standards and Technology) and Micromatter certified materials. Recovery values can vary from 2% for magnesium up to 7% for iron (Debastiani et al., 2021).

### GC–MS analysis

The sample of coal particles (5 g) was subjected to Soxhlet extraction with petroleum ether/methylene chloride (1:1) for 24 h, as described by the EPA method 3540C (EPA 1996). The extract underwent concentration, fractionation using a SiO_2_ column, and analysis through gas chromatography (using an RTX-1MS column from Restek) and mass spectrometry (with 70 eV and m/z 50–450 range in full scan/SIM modes). Capillary column dimensions were 30 m × 0.25 mm (ID) × 0.5 µm (df). The grain size of SiO_2_ was 0.2–0.5 mm, activation temperature and time: 100 °C and 24 h. Chromatographic and spectroscopic data were processed using Thermo XcaliburTM software (Version 2.2 SP1.48, Thermo Fisher Scientific, Inc.) and AMDIS (Automated Mass Spectral Deconvolution and Identification System, Build 130.53, Version 2.70). The saturate and aromatic fractions were separated using hexane and methylene chloride, respectively. GC oven temperature programming was 85 °C (1 min) to 310 °C (5 min) at @ 4 °C/min; split ratio: 5:1; inlet temperature: 250 °C. Helium (99.999%) was used as carried gas:, flow rate: 1.0 mL/min (constant flow).The organic constituents were identified and confirmed by comparing their mass spectra with those available in spectral libraries and databases such as NIST11, NIST Retention Index, and Wiley9. Additionally, certified standard mixtures including C10-C40 alkanes (ref. # 68,281, Sigma-Aldrich) and a calibration mix of PHA (SV Calibration Mix # 6/610 PAHs Mix, cat. # 31,011, Restek) were used for comparison and verification. In addition, the linear temperature-programmed retention indices (R_I_) were calculated for each component and used for comparison purposes with the R_I_ reported in the existing literature (Linstrom & Mallard, 2022).

### Statistical analysis

The normality of the variables was assessed using the Kolmogorov–Smirnov test. To analyze the differences in DNA damage levels between the exposed and control groups, Student's t test was utilized. For the comparison of biomarkers, the nonparametric Mann–Whitney U test was employed. In addition, the concentration of inorganic elements was analyzed by applying the unpaired t test (with Welch's correction) to determine statistical differences between the exposed and control groups. These statistical analyses were conducted using the PRISMA 5.0 software package. To evaluate the relationships between variables and the impact of these parameters on the overall outcomes, principal component analysis based on descriptive data analysis was performed on the damaged marker parameters and inorganic elements. This analysis was carried out using the XLSTAT® 2020.3.1 program (ADDINSOFT SARL, Paris, France) (Addinsoft, 2022).

## Results

The findings from the analysis of visual scores obtained using both the standard comet assay and the FPG-modified comet assay on peripheral blood lymphocytes of the individuals under investigation are presented in Table 1. The table demonstrates a significant elevation in DNA damage levels for both males and females compared to their respective control groups in the standard comet assay (*P* < 0.001). A similar pattern was observed in the modified assay (using FPG enzyme), with noteworthy differences between the exposed and control groups for males and females, respectively (*P* < 0.001). However, the FPG-modified comet assay yielded higher levels of DNA damage (attributable to increased purine oxidation products) compared to the standard assay. Furthermore, statistical significance was also observed when comparing the entire groups (exposed vs. controls) (*P* < 0.001).

**Table 1:** Visual score (0–400; mean ± standard deviation) of peripheral blood lymphocytes from individuals exposed to residual coal dust and controls using the standard and FPG-modified comet assay

Comet assay	Males		Females		Entire group	
	Control (*N* = 50)	Exposed (*N* = 61)	Control (*N* = 70)	Exposed (*N* = 89)	Control (*N* = 120)	Exposed (*N* = 150)
Standard	40.8 ± 15.0	104.4 ± 22.3^a^	48.7 ± 17.4	57.6 ± 18.4^a^	45.4 ± 16.8	76.6 ± 32.8^b^
FPG	43.5 ± 15.3	120.6 ± 22.3^b^	49.9 ± 17.3	68.2 ± 16.0^b^	47.2 ± 16.7	89.5 ± 31.9^b,c^

^a^* P* < 0.01 and ^b^ *P* < 0.001, significant difference in relation to the control groups within the same group (standard or FPG-modified comet assay or entire group); ^c^ *P* < 0.001, significant difference in relation to standard comet assay (entire group)

**Fig. 1:**
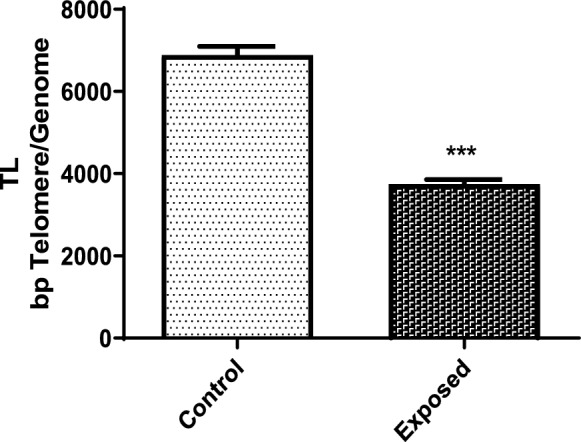
Telomere length (TL) (mean ± standard deviation) in base pairs (bp) for the control and exposed groups. ***Significant difference compared to the control group (*P* < 0.001, Mann–Whitney *U* test)

**Fig. 2:**
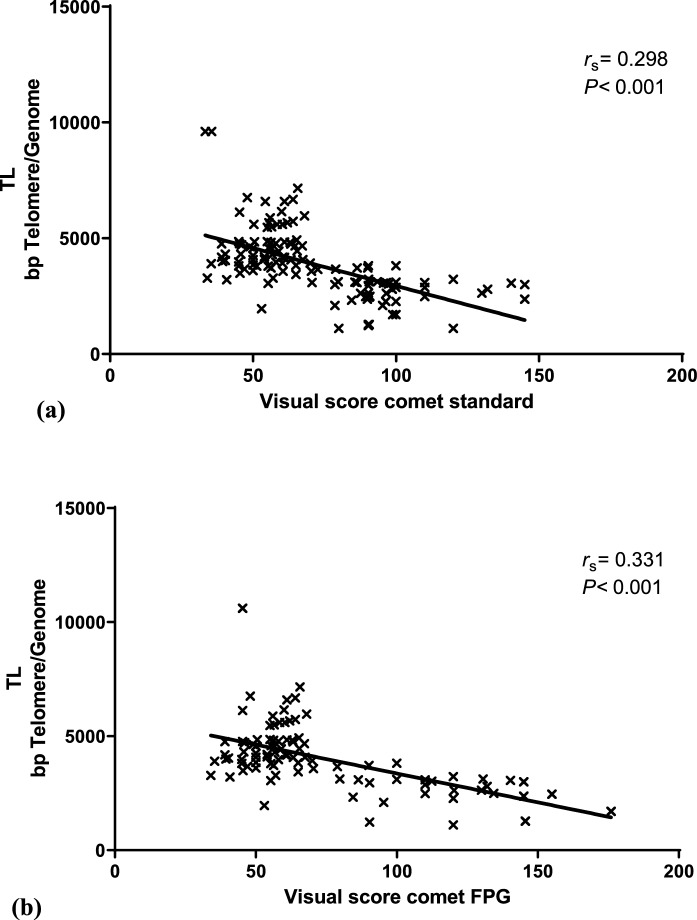
Spearman correlation analysis between telomere length and visual score: **a** standard comet assay and **b** FPG-modified comet assay

The results about TL indicated that the exposed group exhibited significantly shorter TL (3751.0 ± 1315.1) compared to the control group (6891.1 ± 2224.0) (*P* < 0.001, Mann–Whitney U test) (Fig. 1). Moreover, a significant correlation was observed between TL and the visual score obtained from the standard comet assay (*P* < 0.001) (Fig. 2a), as well as the oxidative DNA damage detected through the FPG-modified comet assay (*P* < 0.001) (Fig. 2b). It is noteworthy that individuals with higher levels of oxidative DNA damage had shorter telomeres. Additionally, no significant differences in TL values were observed between males and females (data not shown).

Regarding the quantitative analysis (in µg/mL) of elements in urine samples from both the control and exposed groups using PIXE, the results are presented in Table 2. The analysis successfully identified all eleven expected elements in the samples. Statistical analysis indicated significant differences (*P* < 0.05) in the concentrations of Na, P, S, Cl, K, Fe, Zn, and Br between the exposed and control groups. Furthermore, the control group exhibited significantly higher concentrations of Ca compared to the exposed group (*P* < 0.05). In the exposed group, significant correlations were observed between age and the concentrations of Fe, Na, and P, showing an inverse relationship (Pearson correlation, *P* < 0.05). Additionally, a positive correlation was found between age and Ni levels (Pearson correlation, *P* < 0.05).

**Table 2:** The concentration of inorganic elements in urine (µg/mL; mean ± standard deviation) of the control and exposed individuals to residual coal dust analyzed by the PIXE method

Inorganic elements	Males		Females		Entire group	
	Control (*N* = 50)	Exposed (*N* = 61)	Control (*N* = 70)	Exposed (*N* = 89)	Control (*N* = 120)	Exposed (*N*= 150)
Na	807.9 ± 153.0	752.2 ± 206.9	798.1 ± 120.4	6331.5 ± 3178.5	802.2 ± 134.5	3542.0 ± 3680.4^**a**^
P	665.5 ± 195.3	691.4 ± 186.8	656.2 ± 189.8	1896.1 ± 750.1	660.1 ± 191.4	1293.5 ± 836.0^**a**^
S	676.4 ± 417.1	767.5 ± 184.1	736.5 ± 599.1	6955.6 ± 3900.4	711.2 ± 529.1	3861.5 ± 4277.9^**a**^
Cl	11,112.6 ± 2042.6	11,807.2 ± 2418.3	10,982.7 ± 1937.8	13,600.0 ± 1645.7	11,037.2 ± 1975.0	12,703.5 ± 2176.1^**a**^
K	4846.4 ± 2304.2	5534.3 ± 1762.6	6066.0 ± 3130.2	7174.3 ± 1244.0	5553.5 ± 2866.5	6354.0 ± 1678.8^**a**^
Ca	766.3 ± 193.5	720.7 ± 326.3^**b**^	748.9 ± 193.3	264.4 ± 260.0	756.2 ± 192.8	492.0 ± 365.2^**a**^
Cr	57.8 ± 30.9	57.7 ± 31.4^**b**^	62.9 ± 34.0	30.8 ± 24.9	41.7 ± 30.6	43.9 ± 32.7
Fe	189.4 ± 61.5	191.1 ± 218.4	191.9 ± 65.8	1352.0 ± 680.4	190.8 ± 63.8	771.5 ± 787.4^**a**^
Ni	15.2 ± 3.7	18.0 ± 7.0^**b**^	16.4 ± 3.4	13.6 ± 6.8	15.8 ± 3.5	15.4 ± 7.2
Zn	15.1 ± 6.8	31.5 ± 5.5	14.5 ± 5.9	29.7 ± 4.7	14.7 ± 6.3	30.5 ± 5.1^**a**^
Br	18.9 ± 10.1	19.5 ± 4.6	19.8 ± 10.3	572.8 ± 440.8	19.4 ± 10.2	295.5 ± 434.9^**a**^

^a^Significant difference in relation to the control group (entire group) at *P* < 0.05 (unpaired t test—Welch`s correction); ^b^Significant difference in relation to the female exposed group at *P* < 0.05 (unpaired t test—Welch`s correction); significant difference in bold

**Fig. 3:**
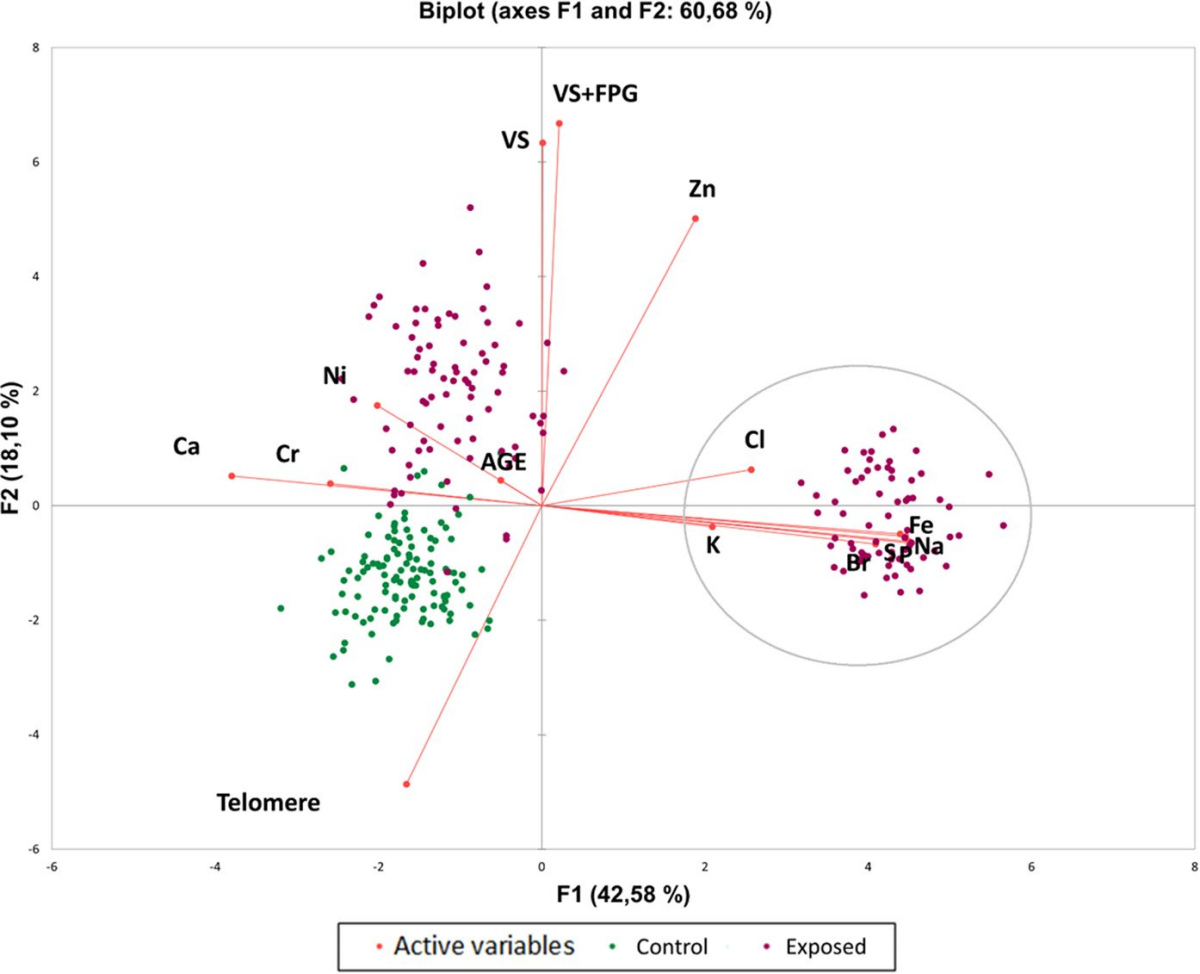
Principle component analysis (PCA) integrating inorganic elements detected in urine samples, DNA damage (VS, visual score using comet assay, with and without FPG; telomere length) from individuals exposed to coal dust and controls. The gray circle highlights the group of exposed women

The results of the principal component analysis (PCA) provided an overview of the distribution of DNA damage and inorganic elements, as well as the relative significance of these data in each sample (Fig. 3). The scores and positions of each sample in the ordering plane (F1 and F2) revealed two significant principal components, which accounted for 42.58% and 18.10% of the total variation, respectively. Consequently, the factor analysis identified two factors responsible for the data structure, explaining 60.68% of the total variance. F1 was positively influenced by damage parameters such as visual score (FPG and basal), as well as the elements Zn, Cl, K, Br, Na, P, S, and Fe. On the other hand, the component F2 was influenced by Ni, Ca, and Cr while being negatively influenced by Telomere. Interestingly, it was observed that men exhibited higher DNA damage (as analyzed by the standard comet assay and FPG enzyme), shorter TL, and higher concentrations of Ni, Ca, and Cr compared to exposed women.

**Fig. 4:**
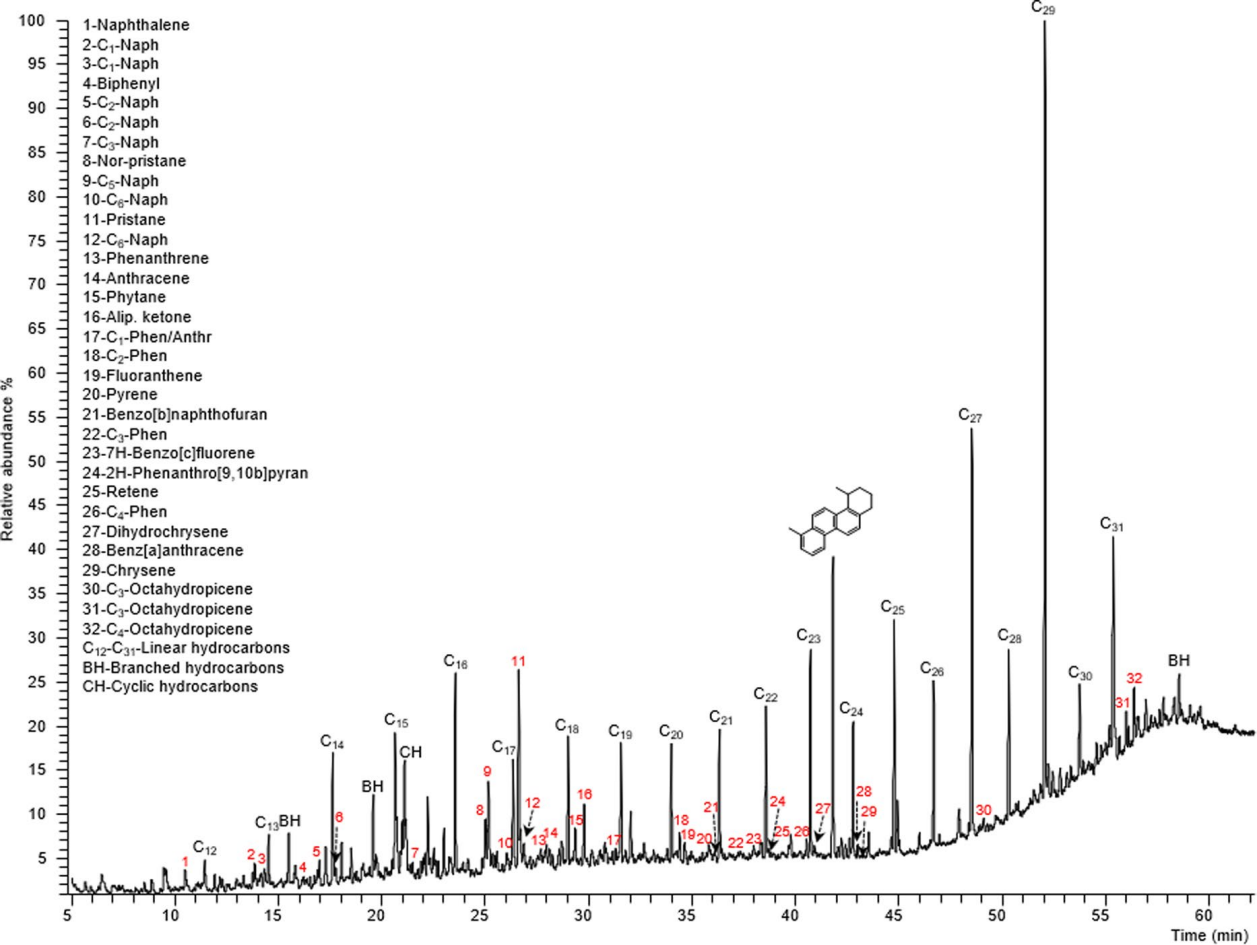
Typical profile (total ion current) obtained by GC–MS (70 eV, full scan) of the total extract from the sample of particulate coal. The names of some relevant constituents (listed according to their order of elution) were included in the Figure, together with C_12_-C_31_ linear, branched, and cyclic hydrocarbons, which were the main components

From the GC–MS analysis (full scan mode) of the organic extract (yield: 0.34%), some relevant constituents (PAC—polycyclic aromatic compounds) were identified together with C12-C31 linear/branched and cyclic hydrocarbons (TIC in Fig. 4). The analysis revealed that the total extract was primarily composed of linear/branched hydrocarbons (51.2%) along with mono-/bicyclic (6.1%) and unsaturated (5.1%) hydrocarbons. Additionally, a diverse group of components with a different chemical nature contributed up to 24.0%, including 4.2% tetracyclic trichromatic hydrocarbons, 3.4% alkyl-naphthalenes, 2.8% alkyl-monoaromatic hydrocarbons, and each 1.8% pentacyclic triaromatic hydrocarbons/PAHs and alkyl-phenanthrenes/anthracenes. Other components included phenolic aldehydes (1.2%), aliphatic ketones (1.1%), aliphatic esters (1.0%), monoaromatic ketones (0.9%), aliphatic ethers (0.6%), and aliphatic di-/trisulfide compounds/phenolic ketones (each 0.5%). Furthermore, the extract contained biphenyls/monoaromatic esters (each 0.4%), benzo[b]naphthofurans/monoaromatic aldehydes/tetracyclic monoaromatic hydrocarbons (each 0.3%), and aliphatic aldehydes/monoaromatic ethers/oxygenated PAHs/quinones (0.2%). Finally, the extract contained oxygenated naphthalenes (0.1%), alkyl pyrenes (0.05%), and alkyl-phenols (0.04%).

**Fig. 5:**
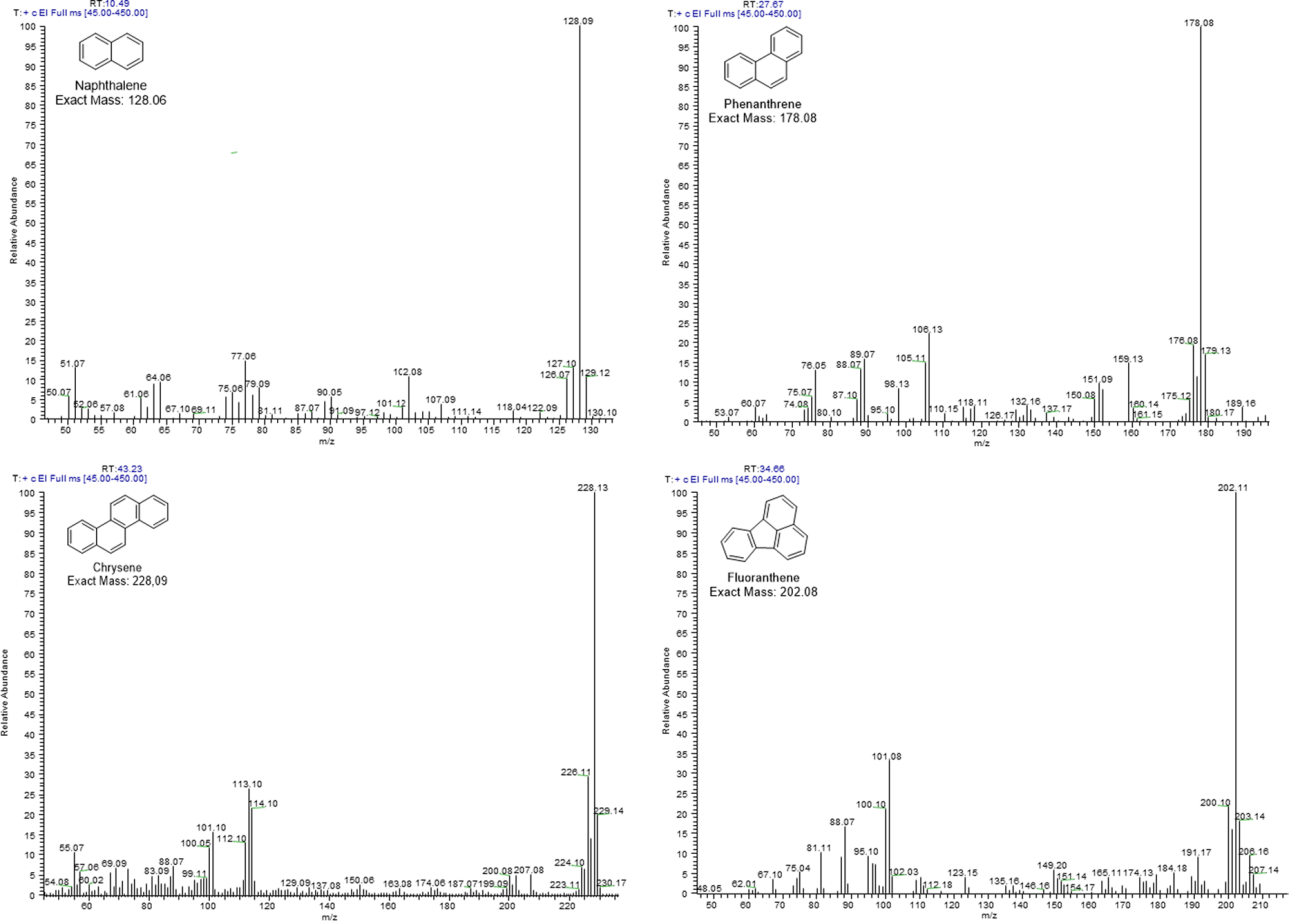
Mass spectra obtained by GC–MS (full scan) of some polycyclic aromatic compounds found in the organic extract from coal sample

From the generalized composition outlined above, more detailed information is given on those constituents that were highlighted: (i) the main component other than linear hydrocarbons was 4,7-dimethyl-1,2,3,4-tetrahydrochrysene (3.5%); (ii) two types of alkyl-naphthalenes were identified: (a) C_1_-C_2_ naphthalenes (1.0%), (b) C_3_-C_8_ naphthalenes (2.4%); (iii) the identified alkyl-monoaromatic hydrocarbons were of two kinds: (a) C_1_-C_5_ benzenes (0.8%), (b) C_6_-C_13_ benzenes (2.0%); (iv) the pentacyclic triaromatic hydrocarbons were derived from 1,2,3,4,4a,5,6,14b-octahydropicene; (v) eight (seven are priority) PAHs were identified (they could be of natural/biogenic/diagenetic or petrogenic origins): naphthalene/fluoranthene (each 0.4%), anthracene/7H-benzo[c]fluorene (each 0.3%), phenanthrene/pyrene (each 0.2%), benz[a]anthracene (0.04%) and chrysene (0.006%); (vi) the alkyl-phenanthrenes/anthracenes were C_1_-C_4_ alkyl-substituted; (vii) the phenolic aldehydes were C_0_-C_2_ alkyl-substituted; (viii) C_12_-C_18_ aliphatic ketones were “2-one” derivatives; (ix) most monoaromatic ketones were alkyl-phenoles substituted; (x) phenolic ketones were C_1_-C_2_ alkyl-substituted; (xi) biphenyls were C_0_-C_4_ alkyl-substituted; (xii) three benzo[b]naphthofuran isomers were identified; (xiii) monoaromatic aldehydes were alkyl-substituted; (xiv) aliphatic aldehydes were all linear; (xv) oxygenated PAHs were derived from 2H-phenanthro[9,10-b]pyran; (xvi) one C_1_ alkyl-pyrene was identified. Figure 5 displays the mass spectra and structures of four of the most important PAHs identified in the coal extract: naphthalene, phenanthrene, chrysene, and fluoranthene.

## Discussion

The role of coal mining in the global economy cannot be ignored, as it has made substantial contributions. However, it is crucial to acknowledge that the pollution generated by coal mining poses a significant threat to public health, as highlighted by studies conducted by Hendryx et al. (2020), Romana et al. (2022), and Kamanzi et al. (2023). In our study, utilizing the standard and FPG-modified comet assay, which is a test employed to detect DNA damage, we observed significant levels of DNA damage in the peripheral blood lymphocytes of individuals who were chronically exposed to coal mining residues. Furthermore, we observed a reduction in TL within the exposed group.

These findings regarding the impacts of coal mining align with the results obtained in studies conducted in various countries, including Sinitsky et al. (2016), Souza et al. (2021), and Ullah et al. (2021). Moreover, similar observations of DNA damage in populations exposed to open-pit coal mining residues have been reported in other regions of Colombia, as demonstrated by studies conducted by Espitia-Pérez et al., 2018a, 2018b. These studies have shed light on the generation of oxidative damage caused by the complex mixture of components released during coal extraction activities. The cumulative effect of oxidative DNA damage is of significant concern, as it has been identified as an underlying factor in numerous diseases. These include neurodegenerative disorders such as Alzheimer's and Parkinson's diseases (Coppedè & Migliore, 2015), autoimmune conditions like rheumatoid arthritis and systemic lupus erythematosus (Souliotis et al., 2019), as well as diabetes (Grindel et al., 2016), cardiovascular diseases (Hu et al., 2021), and cancer (Klaunig et al., 2010; Srinivas et al., 2019).

Significantly, the results of the study unveiled a noteworthy association between DNA damage assessed through the standard comet assay or the FPG-comet assay and the shortening of telomeres. This correlation has been highlighted in various occupational studies, such as those conducted by Guan et al., (2020), Ko et al., (2017); and Li et al., (2015), which have demonstrated the impact of oxidative stress on telomere shortening among exposed populations. Telomere shortening has even been observed in coal mining workers (de Souza et al., 2018) and individuals exposed to indoor air pollution (Lin et al., 2017). Given its significance, TL has been suggested as a potential marker for cancer susceptibility in humans (Heaphy et al., 2022; Ma et al., 2011; Sun et al., 2015).

In this study, we also investigated the factors that could potentially influence the induction of genomic instability in the studied population. Coal has a highly complex chemical composition, containing various amounts of trace elements and metals due to its formation from compressed organic matter that contains almost all the elements of the periodic table (Gopinathan et al., 2022a, 2022b), including heavy metals (Islam et al., 2023; Israr et al., 2022; Song et al., 2022). Coal also contains fly ashes (Panda & Dash, 2020), oxides (Chen et al., 2013; Panda & Dash, 2020), and polycyclic aromatic hydrocarbons (PAHs)(Liu et al., 2008; Ren et al., 2022), among other compounds. The town of La Loma is located approximately 2 km from the nearest coal mine, which may have an impact on certain biomarkers of human health due to exposure to various factors associated with mining activity. These factors can include the emission of coal dust particles, toxic gases, chemicals, and heavy metals, all of which can have harmful effects on the health of individuals exposed to them (Hendryx et al., 2020).

In another study, particulate matter in the air was characterized (León-Mejía et al., 2023b) as an amorphous/irregular solid, with prevailing sizes smaller than 10 µm (PM < 2.5), forming agglomerates that can deposit in different regions of the lung (Darquenne, 2020). For instance, particles smaller than 10 µm are deposited in the alveolar region, where they can remain longer in the lung and cause more inflammatory effects than coarse particles (Schraufnagel, 2020; Shekarian et al., 2021). Various factors play a crucial role in determining the impact of coal and its particulate matter on human health. These factors include chemical composition, charge, surface reactivity, solubility, hydrophobicity, polarity, state of agglomeration, and the ability of the particles to interact with biological tissue and generate ROS (Schraufnagel, 2020).

The samples examined in this study exhibited the presence of inorganic agents associated with oxidative damage. Exposure to coal mining residues can disrupt the balance of inorganic elements, potentially contributing to the observed increase in oxidative damage (León-Mejía et al., 2023b; Souza et al., 2021). The quantitative analysis of inorganic elements in urine, conducted using the PIXE method, revealed elevated concentrations of Na, P, S, Cl, K, Fe, Zn, and Br in the exposed group compared to the control group. Previous studies by León-Mejía et al., (2023a, 2023b) reported significant concentrations of S and Fe in the blood of the exposed population. These elements, present in the inhaled particles, might be absorbed by various tissues and subsequently eliminated through urine (Quintana-Sosa et al., 2021), suggesting a systemic effect on these individuals. Metallic elements can directly interact with organic molecules or indirectly generate ROS, resulting in DNA damage and structural changes in cellular components, thus contributing to the development of various diseases (Chen et al., 2018; Jomova & Valko, 2011).

Our findings revealed an interesting association between age and the urinary concentrations of Fe, Na, and P in individuals exposed to coal mining. As age increased, we observed a decrease in the concentrations of these elements. This observation suggests that chronic exposure to contaminants present in the mining environment may disrupt the homeostasis of Fe, Na, and P in the body. The inverse correlation observed could be attributed to the toxic effects of heavy metals commonly found in coal mining (Islam et al., 2023). Some heavy metals from coal have the potential to interfere with the proper absorption, metabolism, and excretion of Fe, Na, and P, thereby leading to a decline in their urinary concentrations (Jaishankar et al., 2014; Witkowska et al., 2021). Furthermore, a positive correlation between age and Ni concentrations was observed in the group exposed to coal mining. This may indicate that as individuals age, Ni has a progressive buildup in their bodies. This could be due to long-term, chronic exposure to nickel present in the mining environment, either through contaminated air, water, or food intake (Darsow et al., 2012). The correlation could also be influenced by age-related factors, such as a longer lifetime of exposure and a decreased ability of the body to eliminate Ni as it ages (Darsow et al., 2012; Genchi et al., 2020).

It is worth noting that in the obtained results, men exhibited shorter telomeres and higher levels of DNA damage compared to women exposed to coal mining. Men may have a higher susceptibility to the damaging effects of coal mining due to several factors. First, men often occupy physically demanding roles in the mining industry, which may result in increased exposure to hazardous substances and greater inhalation of coal dust. This prolonged exposure can lead to higher levels of toxic substances accumulating in the body, leading to more severe DNA damage (Liu & Liu, 2020; Sinitsky et al., 2016). Secondly, genetic and hormonal differences between men and women may play a role. Some studies suggest that men may have less efficient DNA repair mechanisms or lower antioxidant defenses compared to women, making them more vulnerable to the damaging effects of oxidative stress (Cardano et al., 2022; Fischer & Riddle, 2018). Individual susceptibility to DNA damage and telomere shortening can vary based on genetic factors, overall health status, and additional environmental exposures. Further research is needed to fully understand the specific mechanisms underlying these differences in DNA damage and TL between men and women exposed to coal mining.

Based on the GC–MS chemical analysis of the organic extract from coal, the prominent polycyclic aromatic hydrocarbons (PAHs) identified were naphthalene, phenanthrene, anthracene, fluoranthene, pyrene, benz[a]anthracene, chrysene, and *α*/*β*-methyl naphthalenes. It is important to note that long-term exposure of humans to PAHs through various routes (such as inhalation, ingestion, or dermal absorption) increases the risk of developing different types of cancer (Mallah et al., 2022; Qian et al., 2023). Compounds like benz[a]anthracene (Group 2A) and chrysene are classified by the International Agency for Research on Cancer (IARC) as possibly carcinogenic to humans. Others, including anthracene, fluoranthene, pyrene, and phenanthrene, are categorized under Group 3 due to limited or insufficient experimental evidence on their carcinogenicity in humans (IARC, 2010). PAHs are lipophilic compounds that can easily penetrate cell membranes via passive diffusion after inhalation (Abdel-Shafy & Mansour, 2016). The most plausible mechanism of toxicity (carcinogenicity/genotoxicity) for PAHs in mammals involves their binding with a specific affinity to the aryl hydrocarbon receptor (AHR) (Goedtke et al., 2020), subsequently activating Cytochrome P450 monooxygenases-phase I and other metabolic enzymes (Goedtke et al., 2020; Vogel et al., 2020). This process leads to the formation of oxygenated derivatives (such as diol-epoxides, radical-cations, or redox-active o-quinones), which can form adducts upon reacting with DNA (Moorthy et al., 2015). Numerous studies have demonstrated that PAHs, such as benzo[a]pyrene, fluoranthene, benzo[b]fluoranthene, and phenanthrene, can induce the generation of ROS in different biological systems (Luo et al., 2020; Torres-Ávila et al., 2020).

Furthermore, the aryl hydrocarbon receptor (AHR) plays a crucial role in PAHs metabolism by regulating the expression of genes involved in the initiation, promotion, and progression of various types of cancer (Jenkins et al., 2013; Moorthy et al., 2015; Ren et al., 2022; Tsay et al., 2013).

## Conclusion

Our study revealed an interesting association between age and the urinary concentrations of Fe, Na, and P in individuals exposed to coal mining, suggesting disrupted homeostasis. We also observed a significant correlation between increased DNA damage and oxidative damage with telomere shortening. Furthermore, our findings indicate a progressive accumulation of Ni with age in the exposed group, potentially contributing to the observed DNA damage. Notably, Ni and Cr were found to influence DNA damage in men, while in women, Cl, K, Br, S, P, Fe, and Na were influential factors. These findings provide insights into the complex interplay between exposure to mining contaminants, DNA damage, TL, and the specific elements contributing to the observed effects in both genders. The intricate combination of substances released during coal mining operations has been observed to induce oxidative damage to the DNA of individuals exposed to it. These findings emphasize the risks faced by this susceptible population and emphasize the urgency of implementing new strategies to prevent human carcinogenesis caused by PAHs, inorganic elements, and particulate matter emitted into nearby areas.

## Data Availability

All data generated or analyzed during this study are included in this published article (and its supplementary information files).
